# Mapping the Spatio-Temporal Pattern of the Mammalian Target of Rapamycin (mTOR) Activation in Temporal Lobe Epilepsy

**DOI:** 10.1371/journal.pone.0039152

**Published:** 2012-06-27

**Authors:** Long-Ze Sha, Xiao-Liang Xing, Dan Zhang, Yuan Yao, Wan-Chen Dou, Li-Ri Jin, Li-Wen Wu, Qi Xu

**Affiliations:** 1 National Laboratory of Medical Molecular Biology, Institute of Basic Medical Sciences, Chinese Academy of Medical Sciences and Peking Union Medical College, Beijing, China; 2 Department of Neurology, Sir Run Run Shaw Hospital, ZheJiang University School of Medicine, Hangzhou, China; 3 Department of Neurology, Peking Union Medical College Hospital, Peking Union Medical College, Chinese Academy of Medical Science, Beijing, China; Kaohsiung Chang Gung Memorial Hospital, Taiwan

## Abstract

Growing evidence from rodent models of temporal lobe epilepsy (TLE) indicates that dysregulation of the mammalian target of rapamycin (mTOR) pathway is involved in seizures and epileptogenesis. However, the role of the mTOR pathway in the epileptogenic process remains poorly understood. Here, we used an animal model of TLE and sclerotic hippocampus from patients with refractory TLE to determine whether cell-type specific activation of mTOR signaling occurs during each stage of epileptogenesis. In the TLE mouse model, we found that hyperactivation of the mTOR pathway is present in distinct hippocampal subfields at three different stages after kainate-induced seizures, and occurs in neurons of the granular and pyramidal cell layers, in reactive astrocytes, and in dispersed granule cells, respectively. In agreement with the findings in TLE mice, upregulated mTOR was observed in the sclerotic hippocampus of TLE patients. All sclerotic hippocampus (n = 13) exhibited widespread reactive astrocytes with overactivated mTOR, some of which invaded the dispersed granular layer. Moreover, two sclerotic hippocampus exhibited mTOR activation in some of the granule cells, which was accompanied by cell body hypertrophy. Taken together, our results indicate that mTOR activation is most prominent in reactive astrocytes in both an animal model of TLE and the sclerotic hippocampus from patients with drug resistant TLE.

## Introduction

Temporal lobe epilepsy (TLE) is the most common form of epilepsy in adults, and mesial TLE (mTLE) is often medically intractable [1]–[3]. The majority (approximately 80%) of mTLE patients have seizures that cannot be well controlled with classical anticonvulsant drugs that target ion channels, suggesting that the pathogenesis of mTLE differs from other types of epilepsy [4]. Thus, there is a pressing need to develop new and effective drugs based on our understanding of the molecular mechanisms underlying the epileptogenic process. Many researchers are shifting their focus from membrane proteins to intracellular molecules to find disturbances in signal transduction that may contribute to abnormal neural network activity in TLE [5]. In addition, a number of studies demonstrated that a key role of astrocytes in seizure activity was to drive neurons to seizure threshold [6], [7], and to generate deficits in neuronal inhibition [8], [9]. These findings suggest several novel astrocyte-related targets for drug development.

The mTOR (mammalian target of rapamycin) signaling pathway integrates both intracellular and extracellular signals and serves as a hub regulator of cell growth, proliferation, survival, differentiation, and homeostasis [10], [11]. In the nervous system, mTOR plays important roles in neuronal development, neurite outgrowth and synaptic plasticity [12], [13]. Dysregulation of mTOR signaling has been implicated in various neuropathological disorders including cortical dysplasia, tuberous sclerosis, and neurodegenerative disorders such as Alzheimer’s disease [14].

Recent studies using rodent models of TLE provide evidence that overactivation of the mTOR pathway may contribute to epileptogenesis and spontaneous epileptiform discharges [15], [16]. The first suggestion that mTOR could be a potential therapeutic target for epilepsy came from studies using a mouse model of tuberous sclerosis complex. In these mice, early treatment with rapamycin to inhibit mTOR activation prevents development of epilepsy, and later rapamycin treatment in mice that have already developed epilepsy suppresses seizures [17]. In rodent models of TLE, mTOR is overactivated in hippocampus by chemically-induced seizures, and rapamycin treatment reduces mossy fiber sprouting [18], [19] and inhibits epileptic seizures [20]. Furthermore, a ketogenic diet may be an effective treatment for refractory epilepsy because it inhibits the mTOR pathway [21].

The epileptogenic process involves many pathological changes at the cellular and molecular levels [22], [23], and mTOR pathway is involved in temporal lobe epileptogenesis. Thus, gaining insight into the spatio-temporal pattern of mTOR activation during the onset and progression of epilepsy will increase our understanding of the potential anti-epileptogenic role of mTOR inhibitors. This study aimed to investigate the expression and cellular distribution of phosphorylated S6 ribosomal protein(pS6) on ser235/236, a site well known to be selectively phosphorylated by mTOR and widely used to monitor mTOR activation [11], [24], [25], in both a kainate-induced TLE mouse model and surgical specimens from patients with intractable mTLE.

## Methods

### Ethics Statement

All patients gave written informed consent prior to participation and all procedures in this study were approved by the Ethics Committee of Chinese Academy of Medical Sciences, Peking Union Medical College, and the ethics committee of the Peking Union Medical College Hospital (PUMCH).

All animal work was approved by the Experimental Animal Center of Peking Union Medical College and in accordance with the institutional guidelines of the Beijing Administration Office of Laboratory Animals. All efforts were made to minimize animal suffering and reduce the number of animals used.

### Tissues from Patients with Refractory mTLE and Non-sclerotic Controls

Surgically resected sclerotic hippocampi used in this study were obtained from 18 patients with intractable TLE who underwent surgical treatment at PUMCH. Thirteen patients were diagnosed with hippocampal sclerosis (HS) according to magnetic resonance imaging measurements, electroencephalograohic studies, and histopathologic examination. In the other five patients, focal lesions such as gliomas, cortical dysplasias or oligodendroglioma was observed, but did not involve the hippocampus, and histological examination revealed no significant hippocampal neuronal loss in these patients (non-HS). The non-HS subjects were used as a control group for comparison with the HS cases, similarly to previous studies [26]–[29]. Table 1 summarizes the clinical features of the TLE-HS and non-HS subjects.

**Table 1:** Clinical features of the TLE-HS and non-HS groups. Bold fonts indicate medications taken previously but were ineffective.

TLE-HS	Sex	Age	Time since the first seizure (year)	No. of seizures/month	Medications	Pathology		Early risk factors
1	M	20	5	2.5/M	Topiramate, Valproate, Lamotrigine	HS		no
2	F	33	9	150/M	**Phenobarbital**, **Carbamazepine**, Valproate, Topiramate, Oxcarbazepine	HS		no
3	M	36	12	2.5/M	Carbamazepine, Topiramate	HS		no
4	M	43	13	3.5/M	**Phenytoin**, **Valproate**, **Carbamazepine**, Topiramate	HS		no
5	M	23	13	1.5/M	Valproate, Topiramate, Carbamazepine	HS		Encephalitis B (8 m)
6	F	26	17	15/M	**Carbamazepine**, Valproate, Topiramate	HS		no
7	F	42	23	50/M	**Oxcarbazepine**, Phenytoin	HS		no
8	M	55	43	7.5/M	**Phenobarbital**, **Primidonum**, **Phenytoin**, Carbamazepine, **Nitrazepam**, Topiramate	HS		Pneumonia (6 m)
9	M	46	31	2/M	**Carbamazepine**, Oxcarbazepine	HS		febrile seizures (3 y)
10	M	29	28	6/M	Topiramate, Carbamazepine	HS		febrile seizures (8 m)
11	M	44	14	?	Valproate, **Carbamazepine**, **Nitrazepam**, topiramate, **Phenobarbital**	HS		Meningitis (Childhood)
12	F	23	9	135/M	**Valproate**, **Carbamazepine**, Oxcarbazepine, Topiramate	HS		Neurocysticercosis (9 y)
13	M	20	4	4/M	**Carbamazepine**, Topiramate	HS		febrile seizures (1 y)
				
**non-HS**	**Sex**	**Age**	**Time since the first seizure (year)**	**No. of seizures/month**	**Medications**	**Pathology**	**Early risk factors**	
1	F	33	2 M		Valproate	Glioma	no	
2	M	43	27	14/M	Carbamazepine	Oligodendroglioma	no	
3	M	27	20	10/M	**Phenobarbital**, **Phenytoin**, Carbamazepine	Cortical dysplasia	no	
4	M	29	17	2/M	Topiramate, Lamictal	Glioma	no	
5	M	42	16	5/M	**Carbamazepine**, **Nitrazepam**,**Phenobarbital**, Topiramate,	Glioma	no	

Surgically resected tissues were immediately stored below 10 degrees Celsius and treated with 10% buffered formalin within 1 h after surgery, fixed no later than 48 h, and then embedded in paraffin. Paraffin-embedded tissues were sectioned at 4 µm for immunohistochemistry studies.

### Experimental Animals and Kainic Acid (KA) Induced Mouse Model of TLE

We used adult male C57BL/6 mice (Vital River Animal Technology Co., Ltd., Beijing, China) weighing 20–24 g. For induction of status epilepticus (SE), mice were first anesthetized and stereotaxically injected with 200 ng KA (Sigma, Shanghai, China) in 0.05 µl saline into the right dorsal hippocampus (anteroposterior (AP)  = −2.0, mediolateral (ML)  = −1.8, dorsoventral (DV)  = −2.3 mm), using standard methods [30], [31]. We selected three time points after the KA injection (6 h, 5 days, and 5 weeks) to represent the different stages of epileptogenesis [32]. These time points were also examined in a previous study on mTOR activation in TLE [20]. At least four KA-injected mice were sacrificed at each time point for immunohistochemical evaluation. Control mice were injected with saline alone prior to sacrifice.

### Immunohistochemistry Analysis

KA injected and control mice were deeply anesthetized with pentobarbital, and their brains were rapidly removed, washed with PBS, incubated for at least 48 h in 4% paraformaldehyde (Sigma, ShangHai, China) and paraffin embedded. Paraffin-embedded tissues were sectioned at 4 µm and, mounted on precoated glass slides (GoldenBridge, Beijing, China). Sections with hippocampal structure were used for immunohistochemistry or immunofluorescence studies. The following primary antibodies were used: phospho-S6 ribosomal protein (Ser235/236) antibody (1∶800 in mouse section, 1∶200 in human sections, Cell Signaling Technologies, American, #2211), anti-NeuN (1∶100; Millipore, American, #MAB377), and anti-glial fibrillary acidic protein (GFAP) (1∶500; Abcam, American, #ab4674). Immunohistochemistry was performed using standard methods. After incubating with primary antibody at 4°C overnight, tissue was treated with polymer helper and poly peroxidase-anti-goat IgG (GoldenBridge, Beijing, China) for 10 min each and subsequently incubated in DAB (GoldenBridge, Beijing, China) for the appropriate time. Counterstaining was done with hematoxylin (GoldenBridge, Beijing, China). After each incubation step, sections were washed with TBST (3×5 min).

For immunofluorescence assays, after incubation with the mixed primary antibodies, the slices were incubated with an anti-rabbit secondary antibody conjugated with Alexa488 and Alexa555 (1∶500; Molecular Probes, American) to detect pS6 and GFAP. DAPI (Molecular Probes) was used as a nuclear counterstain. All images were captured using an Axioskop fluorescence microscope (Zeiss, Germany).

### Measurement of Cell Number, Size and Statistical Analysis

**Figure 1:**
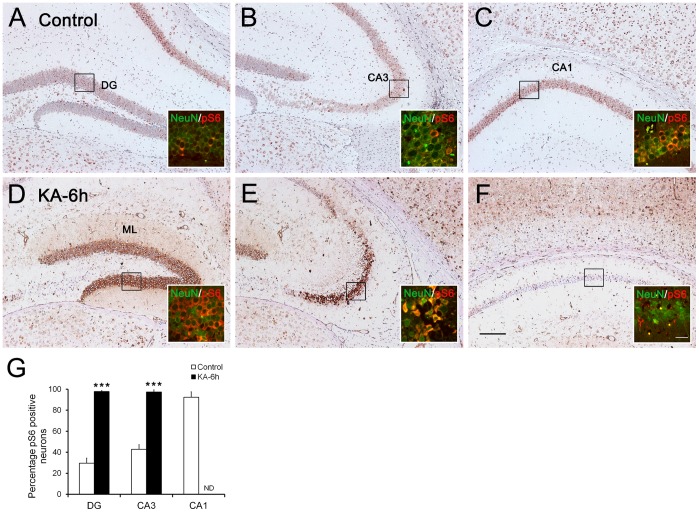
Distribution of pS6 immunoreactivity (IR) in the hippocampus of control mice and 6 hours after induction of seizures. (A-C) In saline-injected control mice (n = 4), the density of pS6 IR varies in the different hippocampal subfields. In dentate gyrus (DG), pS6 IR is weak and only present in some of granule cells (A). In the pyramidal cell layer, pS6 IR is detectable in the entire CA3 subfield (B) and strong in the CA1 region (C). Hippocampus 6 hours post-SE (n = 4) shows significantly increased pS6 IR within the dentate gyrus (D) and CA3 subfields (E) compared with control hippocampus, but pS6 IR is absent in CA1 (F). Insets in D-F show increased expression of pS6 (GFAP-positive, red) in neurons (NeuN-positive, green) of epileptic hippocampus than control hippocampus (A-C). Neuronal death is indicated by nuclear pyknosis. (G) Quantification of pS6 positive neurons in hippocampus at 6 h after seizures (n = 4) versus control mice (n = 4). Error bars are means ± SD., **P<0.01; ***P<0.001 is based on Student’s t-test. ND, not detectable. ML: molecular layer; HS: hippocampal sclerosis; KA, kainic acid. Scale bar in A-F: 200 µm; in insets: 30 µm.

**Figure 2:**
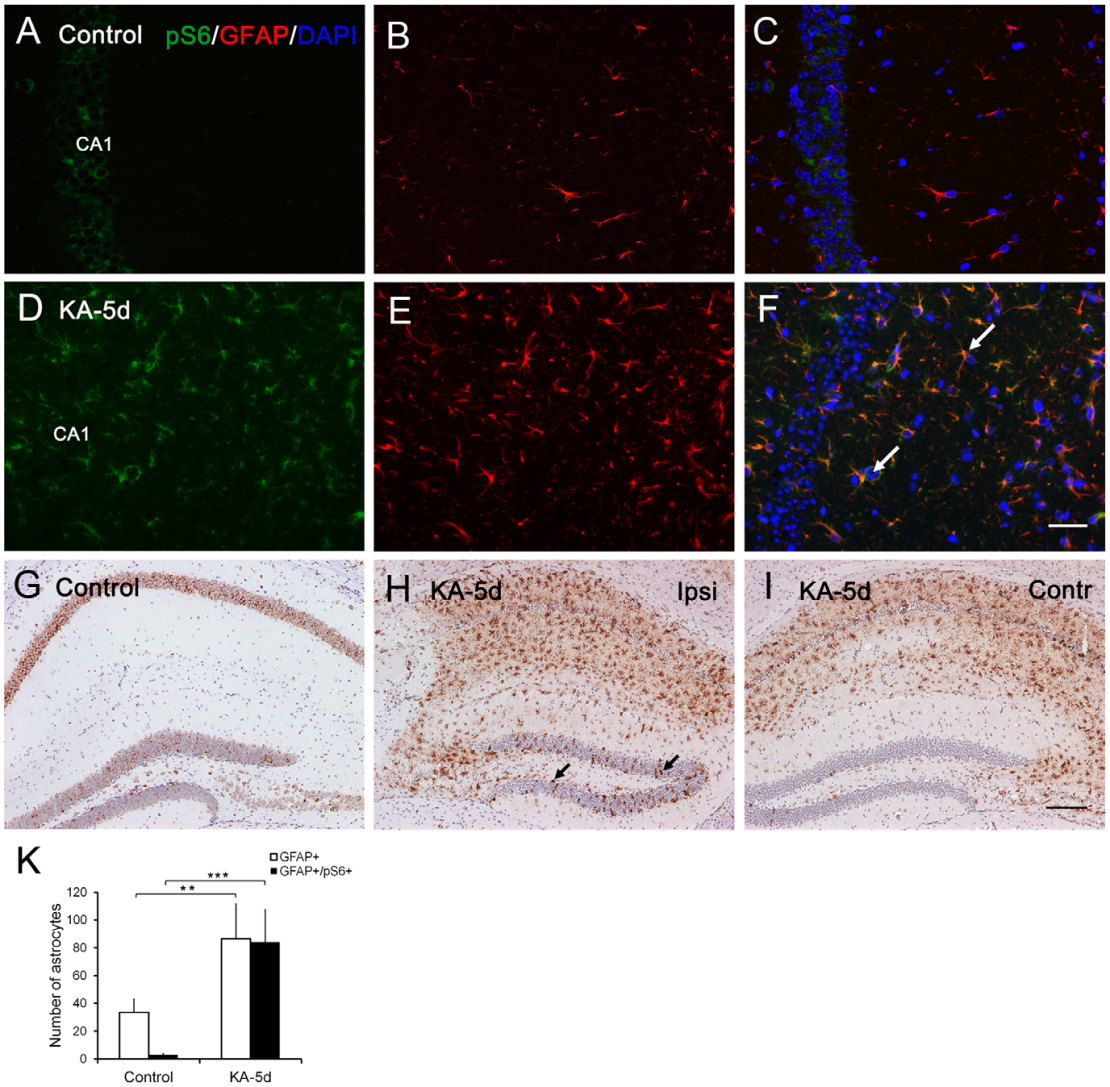
Distribution of pS6 in the hippocampus of control mice and 5 days after induction of seizures. In CA1 of saline-injected control mice (n = 4), pS6 (green) is present in the cytoplasm of pyramidal neurons (A). Immunofluorescent staining of GFAP (red) shows distribution and morphology of astrocytes under normal physiological conditions (B). There is no colocalization of pS6 and GFAP (C). During the latent phase, increased pS6 expression is observed in the CA1 subfield of KA-injected hippocampus (n = 4) (D), and colocalizes with GFAP, mostly in astrocyte-like cells (arrows in F). Reactive gliosis is indicated by dramatically elevated GFAP (E). pS6 positive glia are present in hippocampus both ipsilateral (ipsi, H) and contralateral (contr, I) to KA injection, although elevation of the former is more significant. Compared with saline-injected control hippocampus (G), dentate gyrus of hippocampus ipsilateral to KA injection (H) contains fewer granule cells with strong pS6 IR (arrow in H), while almost no pS6 positive granule cells are present in the contralateral, non-injected hippocampus (I). (K) Quantification of GFAP positive and pS6/GFAP double positive cells in CA1 subfield of KA-injected mice (n = 4) versus control mice (n = 4) at the second stage. Error bars are means ± SD., **P<0.01; ***P<0.001 is based on Student’s t-test. Scale bar in A-F: 100 µm; in G-I: 200 µm.

**Figure 3:**
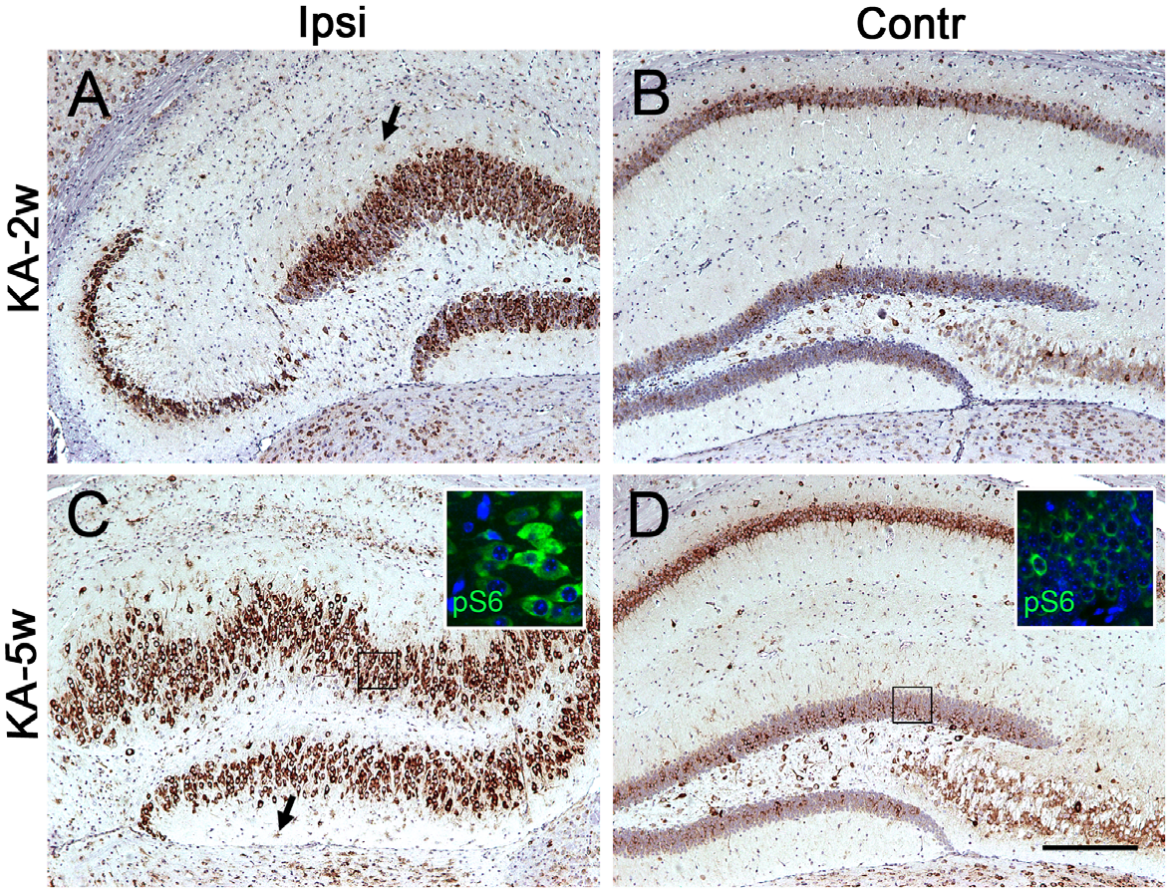
pS6 expression in the sclerotic hippocampus (HS) and non-sclerotic controls (non-HS). Non-sclerotic hippocampus displays weak pS6 IR in glial cells of the hilus (A), while in CA1 and CA3, staining is mainly observed in the cytoplasm of pyramidal cells, and only a few glia are pS6-positive (a-d). In the sclerotic hippocampus, a large number of astrocytes (B) with significantly increased pS6 IR are observed in dentate gyrus (DG) subfields including the hilus (B) and the molecular layer (e). Astrocytes with up-regulated pS6 invade the dispersed granular layer (C). Immunofluorescence image shows colocalization of pS6 (green) with GFAP (red) in astrocytes surrounded by neurons (Inset in C). Glial proliferation and strong pS6 IR are also detected in hilus, CA1 and CA3 of subjects with HS (f-h). (E) Quantification of astrocyte-like pS6 positive cells in CA1, CA3, hilus and DG of HS (n = 13) and non-HS (n = 5). Error bars are means ± SD., *P<0.05; **P<0.01 is based on Student’s t-test. Scale bar in A-B: 400 µm; in C: 50 µm; in D: 20 µm. ML, molecular layer.

After immunofluorescence or immunohistochemistry staining, cell counts were performed on acquired images, by counting cells stained with pS6, GFAP, Neun, pS6/GFAP or pS6/NeuN double-labeled cells. For each mouse or human hippocampus, we analyzed four sections chosen at different levels of anteriority. As shown in Fig. 1, we counted pS6 positive neurons and the numbers of neuron in DG, CA1, CA3, respectively, and calculated the percentage in each of the 3 selected regions. As shown in Fig. 2, GFAP positive and pS6/GFAP double-positive cells were counted in an area of 650 µm x 900 µm in the CA1 subfield. As shown in Fig. 3, we calculated the numbers of astrocyte-like pS6 positive cells in a region shown in Fig. 3D a–h (14400 µm^2^). For each section of human hippocampus, three different areas in each of the 4 selected regions were analyzed (molecular layer of DG, hilus, CA3 and CA1).

**Figure 4:**
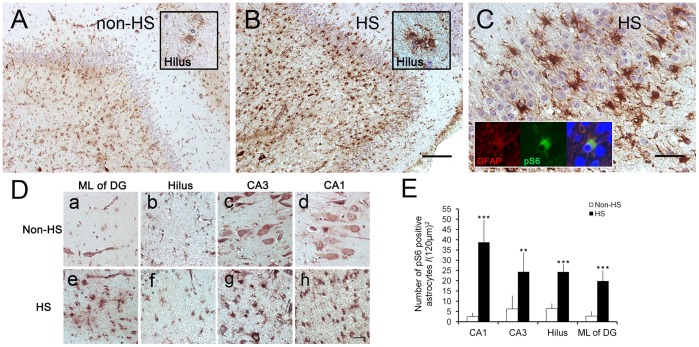
mTOR activation in the granule cells of the sclerotic hippocampus is associated with neuronal hypertrophy. In the dentate gyrus of non-sclerotic hippocampus, granule cells have uniformly sized cell bodies and pS6 expression is below detection level (A). In the sclerotic hippocampus, pS6-positive and negative granule cells are different sizes (B, red and black two-headed arrows indicate pS6 negative and positive neurons, respectively). pS6 positive granule cells in the dentate gyrus have larger cell bodies than do pS6-negative cells (F). A special non-sclerotic hippocampus with recently emerging seizures induced by a glioma also exhibits some granule cells with substantially increased pS6 expression (C), but both pS6-positive and -negative cells have the same cell size (F). KA mice at 2 weeks post-SE were used to study the cell size of pS6 positive and negative neurons, both upper (D) and lower blades (E) of DG were involved in the statistic analysis (F). Error bars are means ± SD., **p<0.01 is based on Student’s t-test. Scale bar in A-C: 50 µm; in D-E: 25 µm.

As shown in Fig. 4, the size of granule cells was determined by measuring across the widest area of cytoplasma (largest possible diameter), usually between the two visible poles of the cell. A similar method has been published elsewhere [33]. For each human subject, we measured at least 60 pS6-positive and -negative cells in 5 different fields of DG. For each KA mouse, we selected 6 circular areas (75 µm diameter) in the upper and lower blades of DG, and measured the size of each type of cells within the circular area. Each circular area contains a center of pS6 negative neurons which were surrounded with pS6/NeuN double-labeled neurons. Student’s t test was used for statistical analysis. The data are presented as mean ± SD.

## Results

To determine the distribution of mTOR activation at different stages of hippocampal lesions, three time points were chosen including 6 hours (the first stage), 5 days (the second stage) and 5 weeks (the third stage) after kainate-induced seizures, which were mainly accompanied by histopathological changes including neuronal death, astrogliosis and granule cell dispersion, respectively.

Firstly, immunohistochemistry assays were performed on mouse brain sections 6 hours after seizure onset. Control hippocampus (n = 4) displayed widespread pS6 immunoreactivity (IR) in neurons of dentate gyrus, CA1 and CA3 (Fig. 1A, B, C). After unilateral intrahippocampal injection of 200 ng KA, mice experienced at least 2 h of convulsive SE, and significantly increased pS6 IR was observed in the dentate gyrus and CA3 regions at 6 h (n = 4, Fig. 1D, E). Extensive neuronal loss in CA1 was indicated by pyknosis and pS6 IR was no longer present in this region (Fig. 1F). In addition, increased pS6 IR was detected in the molecular layer of dentate gyrus (which contains the dendrites of granule cells) (Fig. 1D). Colocalization analysis indicated that pS6 was expressed in NeuN-positive cells (Fig. 1, insets). Quantitative studies confirmed the percentage increase (Fig. 1G).

Previously, pS6 expression levels have been reported to reach a maximum on the fifth day [20]. This stage is characterized by widespread astrogliosis (Fig. 2E). We examined pS6 IR 5 days after KA injection, and found an increase primarily in reactive astrocytes. In saline-injected control hippocampus (n = 4), no pS6 IR could be detected in glial cells (Fig. 2A–C, G). However, at 5 days post-SE, hippocampus both ipsilateral and contralateral to the KA injection site displayed substantially upregulated pS6 IR in the cytoplasm of reactive astrocytes (n = 4), which was accompanied by severe astrogliosis (Fig. 2H, I). Double immunofluorescence verified localization of pS6 in GFAP^-^positive astrocytes in area CA1 (Fig. 2D–F). Levels of pS6 IR in dentate gyrus of ipsilateral (KA-injected) hippocampus were lower than their previously elevated levels during the acute stage, and showed a distribution similar to that observed in control hippocampus which exhibited few neurons with apparent pS6 IR (Fig. 2G, H). In hippocampus contralateral to KA injection, pS6 IR was absent in the entire dentate gyrus (Fig. 2I). Quantitative analysis revealed a significant increase in both GFAP positive and GFAP/pS6 double-positive astrocytes (Fig. 2K).

**Figure 5:**
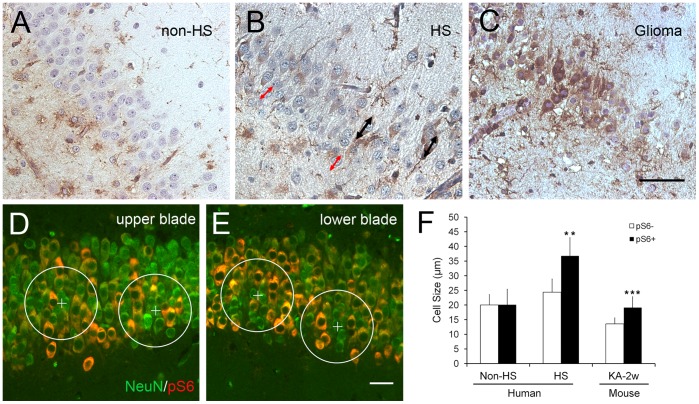
pS6 IR in hippocampus 2 and 5 weeks after KA-induced seizures. (A) Dispersion of granule cells is visible at 2 weeks post-SE ipsilateral (ipsi) to the KA injection (n = 4), (C) and is pronounced at 5 weeks (n = 5). pS6 is significantly upregulated in most of the dispersed granule cells at 2 weeks (A), and in the entire dentate gyrus at 5 weeks (B). The morphology and pS6 IR in the corresponding non-injected (contralateral, conr) hippocampus are normal (B, D). Immunofluorescence for pS6 (green) confirms the same observations. (Insets in C, D). Astrocyte-like cells with increased pS6 IR are widespread in the dentate gyrus and CA area of sclerotic hippocampus (Arrows in A, C). Scale bar in A–B: 400 µm.

Granule cell dispersion is a common pathological feature of patients with mTLE [34], and was reproduced in our mouse model. It also represents the third stage of epileptogenesis. Dispersion was observed at 2 weeks post-SE (n = 4, Fig. 5A), and at 5 weeks it was evident in the entire granular layer (n = 5, Fig. 5C). At 2 and 5 weeks post-SE, the mTOR pathway was activated in dentate granule cells of hippocampus ipsilateral to KA injection (Fig. 5A, C). In addition, pS6 IR in glial cell, while lower at 2 and 5 weeks post-SE than during the second stage, was still higher than that in control hippocampus, and pS6-labeled glial cells were located in the dispersed granular layer and both CA regions (Arrows in Fig. 5A, C). The morphology of the contralateral hippocampus was recovering from excitotoxic injury at 2 and 5 weeks post-SE, and pS6 IR was no longer upregulated (Fig. 5B, D).

Next, we asked whether the observed upregulated mTOR pathway observed in the TLE mouse model also occurs in the sclerotic hippocampus in patients with mTLE. Four non-sclerotic hippocampus exhibited similar distributions of pS6 IR (non-HS No. 2–4). Specifically, astrocyte-like cells with weak pS6 IR were mostly restricted to the hilus (Fig. 3A), while pyramidal neurons in CA1 and CA3 exhibited stronger pS6 IR (Fig. 3,c, d). All sclerotic hippocampus displayed intense labeling for pS6 in reactive astrocytes which were widespread in the dentate gyrus and both CA subfields (Fig. 3B, e–h). Reactive astrocytes with upregulated mTOR not only appeared in non-neuronal layers, but also invaded the dispersed dentate gyrus (Fig. 3C). When compared with the non-HS group, the number of pS6 IR astrocyte-like cells in sclerotic hippocampus was remarkable increased in the molecular layer of DG, in the hilus, in the CA1 and CA3 area, respectively (Fig. 3E).

In the dentate gyrus of four non-HS cases (non-HS No. 2–4), granule cells exhibited uniform cell size and pS6-positive cells were hardly detected (Fig. 4A). The same was true of 11 of the HS cases (HS patients No. 1–11 in Table 1). However, two HS subjects did exhibit a large number of pS6 positive granule cells (Fig. 4B; HS No. 12, 13). One non-HS hippocampus, resected from a patient whose recently emerging seizures were triggered by a glioma, also displayed many granule cells with strong pS6 IR (Fig. 4C; non-HS No.1). We reasoned that the granule cell pS6 IR in the presence of the glioma could have resulted from epileptic discharge, while mTOR activation in granule cells of HS patients in the chronic stage of TLE may be related to neuronal hypertrophy, a hallmark of this disease [35], [36]. To test this idea, we measured the cell size of pS6-positive and surrounding pS6-negative neurons in the dentate gyrus of the two HS and one non-HS (glioma) subjects with elevated pS6 IR. Consistent with our prediction, the cell bodies of the pS6-positive neurons in the two HS subjects were larger than neurons without pS6 IR. However, no difference was found in the size of pS6-positive and -negative granule cells in the non-HS (glioma) subject (Fig. 4F). To reinforce the results, we used KA mice at 2 weeks to ensure whether the cell size is different in pS6 positive versus pS6 negative neurons in the dispersed DG. As mentioned above, the hippocampus exhibited that pS6/NeuN double positive granule cells were surrounded by the NeuN (green) positive neuronal cells at 2 weeks post-SE. Therefore, we selected 6 circular areas in the dentate gyrus of each section (n = 4) which contained both the upper and lower blades (as indicated in Fig. 4D, E), and analyzed the cell size of pS6 positive and negative neurons. The statistical analysis (Fig. 4F) confirmed that mTOR activation in granule cells was accompanied by the increase of cell body size.

## Discussion

Abnormal intracellular signaling in refractory TLE is receiving increasingly more attention and recent studies implicate the mTOR pathway in this disease [15]. In this study, we investigated the spatio-temporal distribution of mTOR activation in an animal model of TLE and in sclerotic hippocampus from patients with drug resistant mTLE. We found that mTOR activation occurs in astrocytes and granule cells in human sclerotic hippocampus, and different implications are associated with each.

### 13/13 HS Patients Exhibited Widespread Reactive Astrocytes with Overactivated mTOR

mTOR activation in the sclerotic hippocampus was most prominent in reactive astrocytes. For obvious reasons, the sclerotic hippocampus can be used to study only the chronic phase of temporal lobe epileptogenesis. Animal models allow investigation of mTOR activation at the two earlier stages of epilepsy [37]. Using a KA-induced TLE mouse model, we found that upregulated mTOR in astrocytes first appeared at the second stage and remained activated at 5 weeks post-SE.

Astrogliosis is a ubiquitous hallmark of all central nervous system pathologies [38], and it has been reported that the mTOR pathway is activated in reactive astrocytes in spinal cord injury [25]. We observed similar mTOR activation in reactive astrocytes of both TLE mice and patients with mTLE.

Previous research reported that mice with conditional deletion of the tuberous sclerosis-1 (TSC-1) gene in astrocytes exhibit abnormal glial proliferation and seizures [39]. Further, astrogliosis can generate deficits in neuronal inhibition [8] and result in reduced astrocytic uptake of potassium and glutamate [6], [38]. Taken together, it raises the possibility that astrocytes with overactivated mTOR could create a microenvironment conducive to the temporal lobe epileptogenic process. Whether it is true needs further study.

A recent study demonstrated that rapamycin treatment (which inhibits mTOR) suppresses mossy fiber sprouting, but does not affect seizure frequency [19]. However, rapamycin treatment has other effects, including inhibition of GABAergic interneurons and changes in synaptic complexity, and these could underlie its failure to suppress seizures completely by blocking mTOR, as the study’s authors discussed. Therefore, targeted inactivation of the mTOR pathway in reactive astrocytes could potentially offer a promising approach to obtain better inhibitory effects in TLE animal model.

### 2/13 HS Patients Displayed mTOR Activation in Granule Cells

Granule cell hypertrophy is a common pathological feature in HS [36]. We found that the mTOR pathway is activated in dispersed granule cells in the third stage of epilepsy in TLE mice, as well as in the sclerotic hippocampus of two patients (HS patients No. 12, 13). An *in vitro* study demonstrated that cell size was controlled by mTOR [40]. Furthermore, increased cell body size of granule cells in the neocortex of patients with TLE has been reported in a previous study [33]. Our results indicated that mTOR activation in granule cells of the sclerotic hippocampus was associated with increased cell body size. The increased size of neurons would yield more surface area for synaptic input [41], a relative constancy in synaptic interval and space between adjacent neurons appears to be important for the homeostasis of neural circuits [42]. Although it has been reported that rapamycin reduced hypertrophy nearly to control levels without affecting seizure frequency in pilocarpine-treated mice [19], it remains open to debate that the side effects of systemic administration of rapamycin might confound the result.

Progressive mTOR activation in granule cells of TLE mice was demonstrated by the increased number of involved granule cells from the early to the late chronic phase (2 and 5 weeks post-SE, respectively). However, only two HS patients exhibited pS6-positive granule cells. We found that both of them had relatively recent onset of TLE (4 and 9 years since their first seizure) compared with most of the other patients studied, suggesting that they were probably in an earlier period of the chronic phase. HS patients No.1 and 2 also had relatively recent TLE yet showed no mTOR activation in dentate granule cells, whether this is due to the clinical heterogeneity require further study based on a larger sample size.

### Non-HS Cases as a Comparison Group

It is now increasingly recognized that abnormalities in cellular signal transduction specific to the sclerotic hippocampus, rather than alterations caused by epileptic discharges, underlie the recurrent seizures seen in HS patients. Therefore, using non-HS cases of TLE as a control group in this study was helpful for determining whether the activated mTOR pathway is responsible for the changes specific to HS. Furthermore, the duration of the post mortem interval might be long enough to lose a substantial proportion of phosphorylated proteins [43], so that non-HS tissues are more suitable for comparison than autopsy tissues due to the collection of both non-HS and HS groups were under the same condition.
